# Phosphorene-Supported Au(I) Fragments for Highly Sensitive Detection of NO

**DOI:** 10.3390/molecules30153085

**Published:** 2025-07-23

**Authors:** Huimin Guo, Yuhan Liu, Xin Liu

**Affiliations:** School of Chemistry, State Key Laboratory of Fine Chemicals, Frontier Science Center for Smart Materials, Dalian Key Laboratory of Intelligent Chemistry, Dalian University of Technology, Dalian 116024, China

**Keywords:** Au, phosphorene, NO, NO_x_ detection, single-site heterogeneous catalysis

## Abstract

The fabrication and application of single-site heterogeneous reaction centers are new frontiers in chemistry. Single-site heterogeneous reaction centers are analogous to metal centers in enzymes and transition-metal complexes: they are charged and decorated with ligands and would exhibit superior reactivity and selectivity in chemical conversion. Such high reactivity would also result in significant response, such as a band gap or resistance change, to approaching molecules, which can be used for sensing applications. As a proof of concept, the electronic structure and reaction pathways with NO and NO_2_ of Au(I) fragments dispersed on phosphorene (Pene) were investigated with first-principle-based calculations. Atomic-deposited Au atoms on Pene (Au_1_-Pene) have hybridized Au states in the bulk band gap of Pene and a decreased band gap of 0.14 eV and would aggregate into clusters. Passivation of the Au hybrid states with -OH and -CH_3_ forms thermodynamically plausible HO-Au_1_-Pene and H_3_C-Au_1_-Pene and restores the band gap to that of bulk Pene. Inspired by this, HO-Au_1_-Pene and H_3_C-Au_1_-Pene were examined for detection of NO and NO_2_ that would react with -OH and -CH_3_, and the resulting decrease of band gap back to that of Au_1_-Pene would be measurable. HO-Au_1_-Pene and H_3_C-Au_1_-Pene are highly sensitive to NO and NO_2_, and their calculated theoretical sensitivities are all 99.99%. The reaction of NO_2_ with HO-Au_1_-Pene is endothermic, making the dissociation of product HNO_3_ more plausible, while the barriers for the reaction of CH_3_-Au_1_-Pene with NO and NO_2_ are too high for spontaneous detection. Therefore, HO-Au_1_-Pene is not eligible for NO_2_ sensing and CH_3_-Au_1_-Pene is not eligible for NO and NO_2_ sensing. The calculated energy barrier for the reaction of HO-Au-Pene with NO is 0.36 eV, and the reaction is about thermal neutral, suggesting HO-Au-Pene is highly sensitive for NO sensing and the reaction for NO detection is spontaneous. This work highlights the potential superior sensing performance of transition-metal fragments and their potential for next-generation sensing applications.

## 1. Introduction

Nitrogen oxides (NO_x_), represented by NO and NO_2_, are harmful to both the living environment and human health. Considerable research attention has been devoted continuously to develop novel procedures and devices for efficient and sensitive detection of NO_x_ [1,2,3,4,5]. Two-dimensional (2D)-material-based NO_x_ sensors are emerging for the large surface area, easiness of functionalization, superior electronic and optical properties, etc., of the materials [6,7,8,9,10,11] and are complementary to conventional metal-oxide-based and metal-nitride-based sensors, etc., in NO_x_ detection [12,13].

Phosphorene (Pene), also known as single- or few-layer black phosphorus, is one of the most outstanding 2D materials since the rise of graphene [14,15,16,17]. In Pene, P atoms are covalently interconnected into 2D layers, and these layers are stacked together through van der Waals interactions. Compared with other 2D materials, Pene possesses a tunable direct band gap (from 0.3 to 2.0 eV, depending on its thickness) and a high carrier mobility (approximately 1000 cm^2^/V·s at r. t.), making it an excellent candidate for gas-sensing applications [18]. The gas-sensing application of Pene has drawn considerable research attention in recent years. Cho et al. investigated the sensing performances of Pene, MoS_2_, and graphene and found that the performance of Pene is superior in dynamic sensing response, sensitivity, selectivity, and response time. They showed that the sensitivity of Pene to target gases are ~20 times higher than those of graphene and MoS_2_. The response time of Pene was ~40 times shorter than that of other 2D materials capable of ppb-level gas detection [19]. Cui et al. fabricated a phosphorene-based sensor and demonstrated that this sensor could detect NO_2_ at ppb levels in dry air at room temperature [20]. Zhang et al. investigated the adsorption of NO, NO_2_, CO, CO_2_, and NH_3_ on Pene under different strains with first-principle-based calculations and showed that the adsorption energies depend strongly on the charge transfer with Pene [21]. Kumawat et al. explored the potential of armchair Pene nanoribbons for explosive detection through first-principle-based calculations and found that these nanoribbons exhibit excellent sensitivity and selectivity toward certain explosive molecules [22]. Apart from being used directly, Pene can be modified further for sensing applications. Surface modification of Pene is primarily achieved through the generating of surface defects or deposition of transition-metal species [23,24,25]. Doping with transition metals [26,27,28,29] or compounds [30,31,32] would not only benefit the air and humidity stabilities and oxidation resistance of Pene [33,34] but also significantly enhance the gas-sensing performance of Pene [35,36,37,38,39,40,41,42]. Cheng et al. proposed that Ag- and Au-doped Pene would be highly sensitive for NO sensing according to the change of band gap [29]. Ghadiri et al. investigated the H_2_S-sensing performance of pristine Pene and Mn-modified Pene by first-principle-based calculations and showed that Mn-modified Pene is highly selective to H_2_S and is reusable [43]. Ghambarian et al. investigated the H_2_-sensing performances of Ni-, Pt-, and Pd-doped Pene and showed that Ni-doped Pene can be used for H_2_ purification, while Pt-doped Pene can be used as a H_2_ sensor [44]. Wang et al. reported the synthesis and NO_2_-sensing application of Ag-nanoparticle-modified Pene [45]. To this end, surface modification with various metal fragments would result in transition-metal-Pene-based sensors with improved gas-sensing performance compared with pristine Pene [46]. Furthermore, the vast combinations of transition-metal fragments on Pene also enable the simultaneous detection and identification of different target molecules [47]. However, it should be noted that most of the aforementioned detection is based on the adsorption of target molecules onto the reactive sites of the senor. The undercoordination of transition-metal atoms on Pene also enables them to adsorb and activate approaching molecules efficiently at the expense of selectivity.

The fabrication and application of single-site heterogeneous reaction centers are new frontiers in chemistry. Single-site heterogeneous reaction centers are analogous to metal centers in enzymes and transition-metal complexes; they would exhibit superior reactivity and selectivity in chemical conversion. Their performance would be tunable by controlling the charge and ligands on them, and they are different from conventional singlet atom sensors. The high reactivity would also result in significant response, such as band gap or resistance change, to approaching molecules, which can be used for sensing applications. Inspired by the superior selectivity of single-site heterogenous reaction centers and the reported sensing performance of Pene-based sensors, we investigated the electronic structure and reaction pathways with NO and NO_2_ of Au(I) fragments on Pene with extensive first-principle-based calculations [46,48,49]. We expect these Au fragments to exhibit superior sensing performance to NO and NO_2_ in terms of high reactivity, selectivity, and fast response. We also expect the findings to pave the way for design and fabrication of Pene-based high-performance sensors for NO_x_ detection.

## 2. Results and Discussions

**Figure 1:**
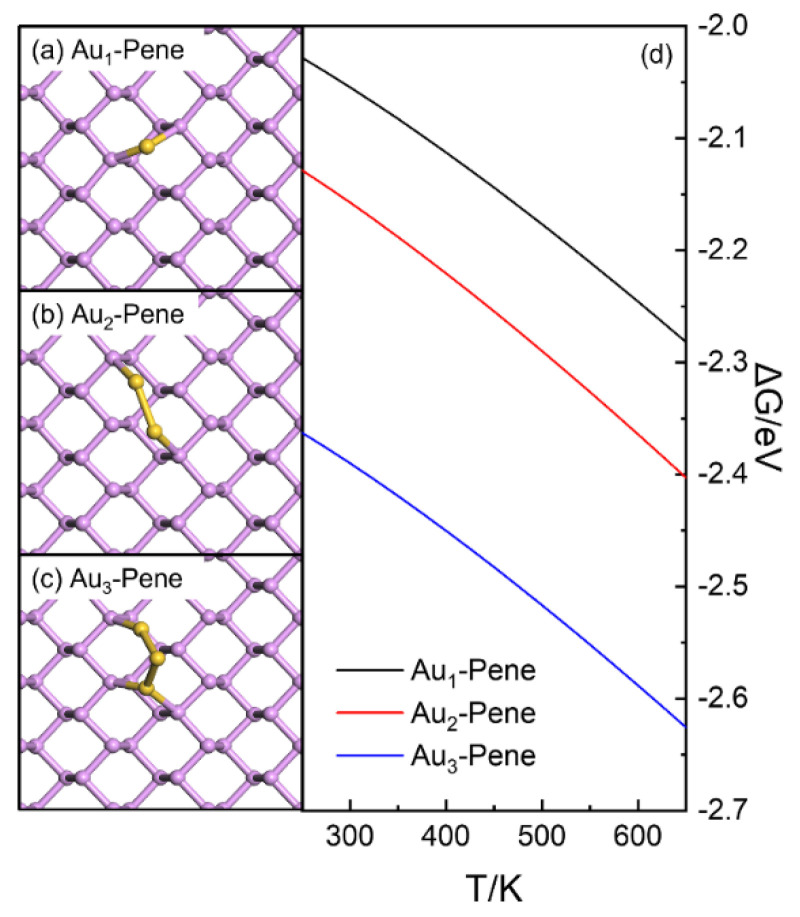
Structures (**a**–**c**) and energetic properties (**d**) of Au_1_ (**a**), Au_2_ (**b**), and Au_3_ (**c**) clusters deposited on Pene. In (**a**–**c**), P and Au atoms are in purple and gold, respectively.

The adsorption structures of Au atoms and small clusters containing two and three Au atoms on Pene were investigated firstly (Figure 1). The Au adsorption on top of a surface P atom (T), bridging two nearest neighboring P atoms (B), and on top of the center of three surface P atoms (H) were investigated, and all potential spin states were considered. The adsorption of a Au atom at B site is found plausible, and the calculated adsorption energy (E_b_) is −1.48 eV, corresponding to a doublet spin symmetry (Au_1_-Pene, Figure 1a). This is in reasonable agreement with the reported value of −1.61 eV for Au atomic adsorption on Pene obtained with VASP [50]. The slight difference can be attributed to the different implementation of theory within the code used. The adsorption of Au_2_ and Au_3_ clusters was also investigated in the same way (Figure 1b,c). The calculated E_b_ values averaged over the number of Au atoms are −1.61 and −2.06 eV for Au_2_-Pene and Au_3_-Pene, respectively, and the calculated free–energy values of these Au species follow the same trend in the T range from 250 to 600 K (Figure 1d). In this sense, Au_1_-Pene on Pene is not thermodynamically stable, and Au atoms would aggregate to form clusters [51]. This is different from the previously reported case of Pd atomic deposition on Pene, where Pd_1_-Pene is preferred [52].

**Figure 2:**
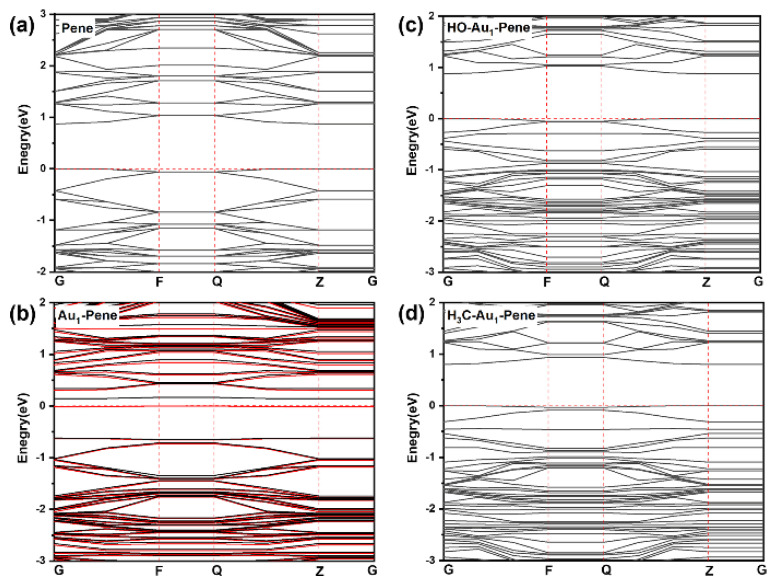
Band structure of pristine Pene (**a**), Au_1_-Pene (**b**), HO-Au_1_-Pene (**c**), and H_3_C-Au_1_-Pene (**d**). In (**a**–**d**), energy “0” corresponds to calculated Fermi level of the system. In (**b**), the bands of spins are in red and black, respectively.

The calculated band gap of pristine Pene is 0.87 eV [48,52,53]. The highest occupied state of Au_1_-Pene is the half-occupied localized state on the Au atom and is within the band gap of Pene at the Fermi level. The calculated band gap of Au_1_-Pene is only 0.14 eV and is much lower compared with pristine Pene. According to molecular orbital theory, the chemical bonding of Au with a species with a half-occupied state would pair the electrons and downshift this Au state, leading to a drastically enlarged band gap that is similar to that of bulk Pene. The passivation of the state on Au with -OH(HO-Au_1_-Pene) and -CH_3_(H_3_C-Au_1_-Pene) were investigated as model systems (Figure 2c,d). With the passivation of the unpaired electron state on Au_1_-Pene, the band gaps of HO-Au_1_-Pene (Figure 2c) and H_3_C-Au_1_-Pene (Figure 2d) increase to 0.88 and 0.80 eV, respectively, as expected. The ground states of these Au(I) fragments on Pene have singlet symmetry. Such change in electronic structure can be considered the response of Au_1_-Pene to the stimuli induced by the adsorption or bonding of the approaching -OH and -CH_3_. The band gap change is ~0.74 eV, which would be significant enough to be detected. However, as Au_1_-Pene is a highly reactive radical with the unpaired electron localized on the Au atom and we have shown that Au_1_-Pene would aggregate to form plausible Au clusters on Pene, it cannot be used directly to detect NO_x_. HO-Au_1_-Pene (Figure 2c) and H_3_C-Au_1_-Pene (Figure 2d) were examined further, considering their potential reactions with species with unpaired electrons, such as NO_x_, etc., may generate more plausible compounds, leaving the band gap decreasing to ~0.14 eV in formed Au_1_-Pene.

**Figure 3:**
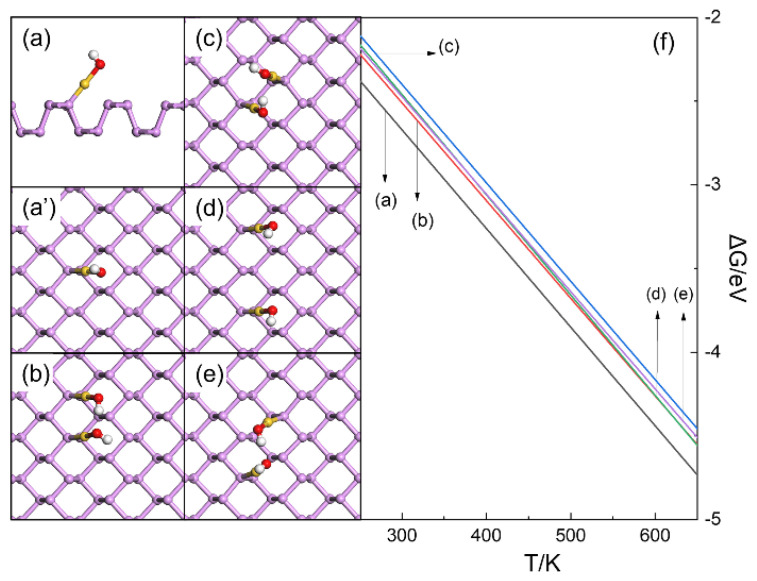
Side (**a**) and top (**a’**) views of structure HO-Au_1_-Pene, top view of structures of potential dimers of HO-Au_1_-Pene (**b**–**e**), and formation free energy (**f**) of HO-Au_1_-Pene and its potential dimers. In (**a**–**e**), P, Au, O, and H atoms are in purple, gold, red, and white, respectively.

**Figure 4:**
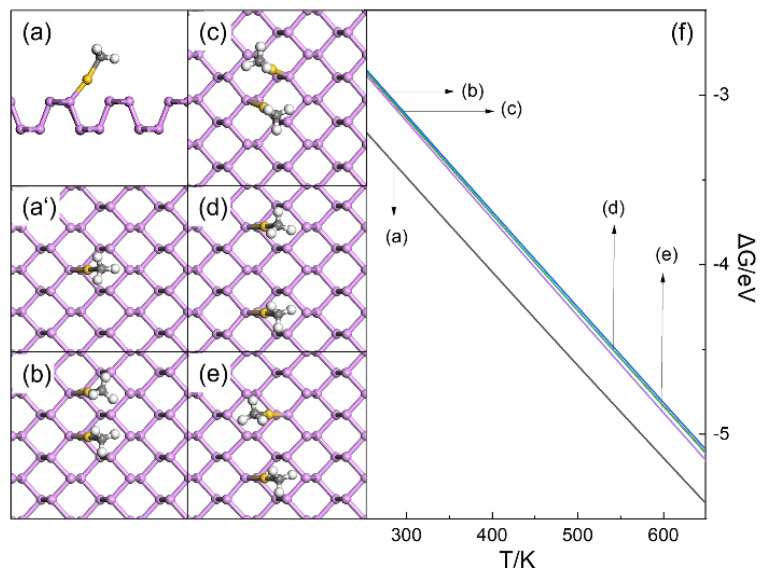
Side (**a**) and top (**a’**) views of structure H_3_C-Au_1_-Pene, top view of structures of potential dimers of H_3_C-Au_1_-Pene (**b**–**e**), and formation free energy (**f**) of H_3_C-Au_1_-Pene and its potential dimers. In (**a**–**e**), P, Au, C, and H atoms are in purple, gold, gray, and white, respectively.

The possibilities for the clustering of HO-Au_1_-Pene and H_3_C-Au_1_-Pene were examined firstly (Figure 3 and Figure 4). After passivation with -OH, the Au atom is at its +1 valence state and coordinates linearly with -OH and one surface P atom (Figure 3a,a’). If the Au(I) fragments are far apart, their contribution to the electronic structure would be the same as a single Au(I) fragment. The possible dimers of HO-Au_1_-Pene were investigated, and hydrogen bonds between adjacent Au(I) fragments are apparent (Figure 3b–d). The calculated formation free energy (ΔG) in Figure 3f shows that these hydrogen bonds are not strong enough to contribute to additional stability of the dimers, and the monodispersed HO-Au_1_-Pene is the most plausible among all the structures considered (Figure 3f). The same was found for H_3_C-Au_1_-Pene (Figure 4), where the Au atom is also +1 |e| charged and coordinates linearly with both -CH_3_ and a surface P atom in the most plausible monodispersed form (Figure 4a,a’). As there are only van der Waals interactions between H_3_C-Au_1_-Pene and the dimers (Figure 4b–e), the difference in formation free energy between the monomer and the dimer is ~0.3 eV (Figure 4f) and is even larger than that for dimers of HO-Au_1_-Pene. Based on these, it can be safely concluded that monodispersed Au(I) fragments are plausible when they are far apart and there is no risk for them to aggregate into clusters.

**Figure 5:**
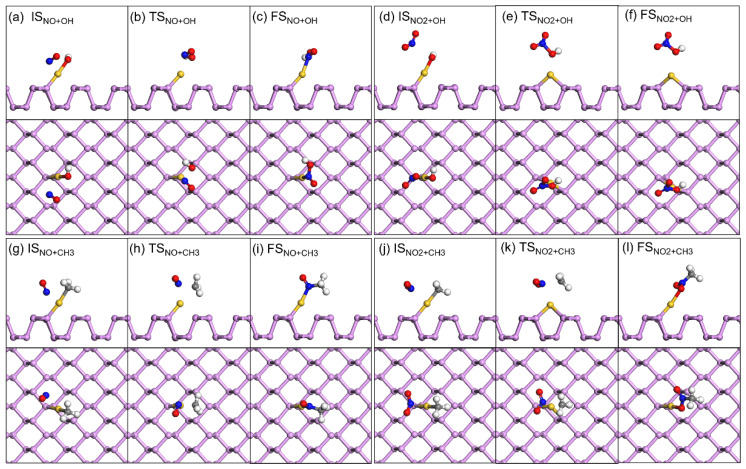
Side view (top panel) and top view (lower panel) of species involved in reactions between NO and HO-Au_1_-Pene (**a**–**c**), NO_2_ and HO-Au_1_-Pene (**d**–**f**), NO and H_3_C-Au_1_-Pene (**g**–**i**), and NO_2_ and H_3_C-Au_1_-Pene (**j**–**l**). In (**a**–**l**), species named IS and FS are the initial and final species involved in the elementary reactions and species named TS are the corresponding transition states. In (**a**–**l**), the N, P, C, O, Au and H are in blue, purple, gray, red, yellow, and white, respectively.

**Figure 6:**
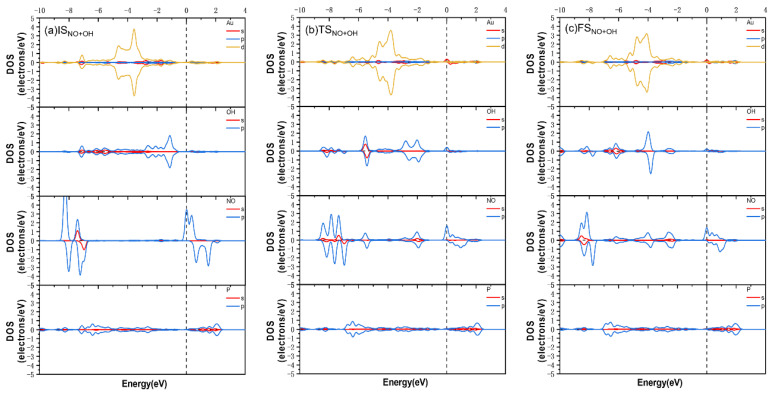
PDOS of IS_NO+OH_ (**a**), TS_NO+OH_ (**b**), and FS_NO+OH_ (**c**).

**Figure 7:**
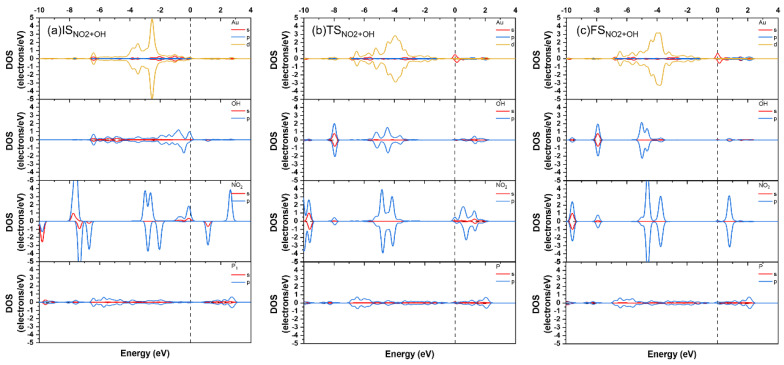
PDOS of IS_NO2+OH_ (**a**), TS_NO2+OH_ (**b**), and FS_NO2+OH_ (**c**).

**Figure 8:**
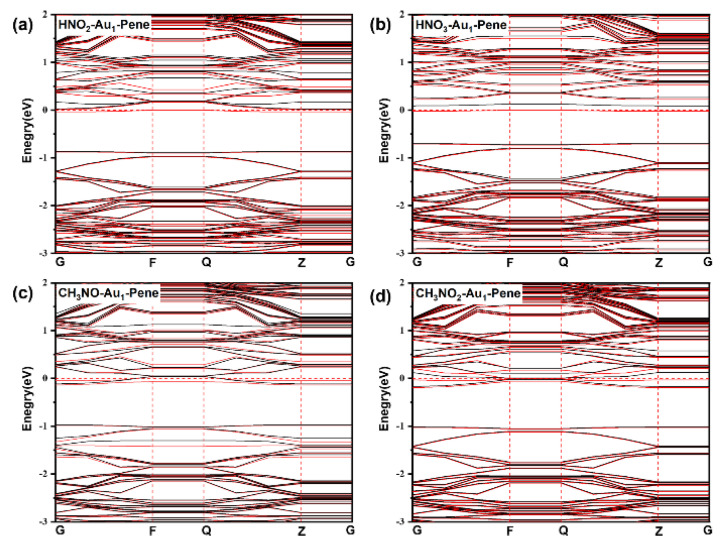
Band structure of pristine HNO_2_-Au_1_-Pene (**a**), HNO_3_-Au_1_-Pene (**b**), H_3_CNO-Au_1_-Pene (**c**), and H_3_CNO_2_-Au_1_-Pene (**d**). In (**a**–**d**), energy “0” corresponds to calculated Fermi level of the system, and the bands of spins are in red and black, respectively.

The mechanistic pathways for the reactions between NO and NO_2_ and HO-Au_1_-Pene and H_3_C-Au_1_-Pene were investigated to highlight the potential sensing performance of these Pene-supported Au(I) fragments (Figure 5). NO would adsorb on and react with HO-Au_1_-Pene spontaneously, forming HNO_2_ adsorbed on Au_1_-Pene by crossing TS_NO+OH_, and the calculated energy barrier and reaction heat are 0.36 and 0.01 eV, respectively. During this process, the N-O distance increases from 1.16 Å in IS_NO+OH_ (Figure 5a) gradually to 1.18 Å in TS_NO+OH_ (Figure 5b) and finally to 1.20 Å in the formed HNO_2_ in FS_NO+OH_ (Figure 5c). Correspondingly, the Au-N distance is 2.73 Å in IS_NO+OH_ (Figure 5a), which decreases to 2.29 Å in TS_NO+OH_ (Figure 5b) and further to 2.16 Å to form adsorbed HNO_2_ in FS_NO+OH_ (Figure 5c). The changes in N-O and Au-N distances can be attributed to the formation of Au-N and N-OH bonds. During the reaction, the Hirshfeld charge on Au decreases from 0.16 |e| in IS_NO+OH_ to 0.12 |e| in FS_NO+OH_, and the Hirshfeld charge on N increases from 0.02 |e| in IS_NO+OH_ to 0.07 |e| in FS_NO+OH_, indicating Au(I) is reduced and N is oxidized, and this is in accordance with the proposed mechanism that the Au(I) fragment is reduced to Au_1_-Pene and NO is oxidized into HNO_2_ adsorbed with N on Au_1_-Pene. These findings are further supported by the DOS analysis (Figure 6 and Figure 7). In IS_NO+OH_, the adsorption of NO is not strong, the molecular states of NO are apparent, and the spin is mainly localized on NO (Figure 6a). At the corresponding transition state, TS_NO+OH_, NO is approaching the HO-Au_1_-Pene, so the spin is no longer localized only on NO but also on the sp states of OH and dsp states of Au (Figure 6b). The newly appeared resonance of occupied dsp hybridized states of Au and NO states at the Fermi level indicates the formation of a Au-N bond and the reduction of Au (Figure 6b,c). This, together with the shortened Au-N distance, explains the adsorption of HNO_2_ (E_ads_: −0.55 eV). The downshift of -OH sp states and NO states also suggests the plausible formation of N-OH (Figure 6b) and the oxidation of N. Due to the bonding and charge transfer between HNO_2_ and Au, the spin is delocalized on Au and HNO_2_ in the product (Figure 6c). The calculated band gap of HNO_2_ adsorption structure is 0.06 eV, confirming the adsorption and reaction of NO would lead to a detectable band gap change of 0.81 eV (Figure 8a).

During the reaction of adsorbed NO_2_ with HO-Au_1_-Pene (IS_NO2+OH_, Figure 5d), NO_2_ would move to attack the O of the OH attached to Au, with the insertion of NO_2_ into the Au-OH by crossing TS_NO2+OH_ (Figure 5e) to form a HNO_3_ adsorbed on Au_1_-Pene (FS_NO2+OH_, Figure 5f), and the calculated energy barrier and reaction heat are 0.77 and 0.61 eV, respectively. In the NO_2_ adsorption structure (IS_NO2+OH_), the Au-O(H) and N(O_2_)-O(H) distances are 2.02 and 2.82 Å, respectively. The sharp spikes of NO_2_ states are apparent, and only the DOS of NO_2_ and OH are spin polarized in Figure 7a, suggesting limited interaction between NO_2_ and HO-Au_1_-Pene. The approaching of NO_2_ leads to the increase of Au-O(H) distance to 2.40 Å at TS_NO2+OH_ and the decrease of N(O_2_)-O(H) distance to 1.63 Å, showing the tendency for the breaking of the Au-O bond and the formation of the N-OH bond. This is further supported by the downshift of DOS peaks of both OH and NO_2_ states and the resonance among Au, OH, and NO_2_ states in the range from −8 to −3 eV (Figure 7). The hybridized states of Au resonate with that of NO_2_ at the Fermi level, confirming the tendency for the insertion of NO_2_ into Au-OH (Figure 7b). In the product of this step, the Au-O(H) distance increases to 2.83 Å and the N(O_2_)-O(H) distance decreases further to 1.47 Å, showing the Au-O bond is broken and the OH is attached to NO_2_, forming a HNO_3_ adsorbed on Au. This process is also accompanied by charge transfer. The Hirshfeld charges on Au and N change from 0.14 and 0.16 |e|, respectively, in IS_NO2+OH_, to 0.10 and 0.27 |e|, respectively, in FS_NO2+OH_, indicating that the Au species is reduced by the charge transferred from the N of NO_2_ that becomes oxidized. The DOS spikes of the NO_2_ and OH states are sharp and well separated and resonate in the range from −10 eV to the Fermi level, confirming the plausible formation of HNO_3_. A new spin-polarized hybridized Au state emerges at the Fermi level, confirming Au gains charge and is reduced during this process and the spin is localized on Au in FS_NO2+OH_ (Figure 7c). To this end, the large barrier at TS_NO2+OH_ can be attributed to the direction of charge transfer from NO_2_ to Au. The calculated band gap of the HNO_3_ adsorption structure is 0.09 eV, confirming the adsorption and reaction of NO_2_ would lead to a detectable band gap change of 0.80 eV (Figure 8). However, the endothermicity and the low reverse-reaction barriers make HO-Au_1_-Pene less eligible for NO_2_-sensing applications.

The reactions between NO and NO_2_ and H_3_C-Au_1_-Pene were also investigated (Figure 5g–l). The calculated energy barriers for the formation of H_3_CNO-Au_1_-Pene and H_3_CNO_2_-Au_1_-Pene are 1.53 and 1.95 eV, respectively; the calculated reaction heat are −0.48 and −0.46 eV, respectively; and the corresponding band gap changes are 0.75 and 0.79 eV, respectively (Figure 8c,d). Though exothermic, the energy barriers are too high for the reactions between NO and NO_2_ with H_3_C-Au_1_-Pene to take place spontaneously. Therefore, H_3_C-Au_1_-Pene is not eligible for NO_x_-sensing applications.

For comparison, the adsorption and reaction of O_2_ and H_2_O on HO-Au_1_-Pene were also investigated. H_2_O would form a hydrogen bond with the -OH moiety of HO-Au_1_-Pene, and the calculated adsorption energy is −0.30 eV, which is slightly more plausible than that on pristine Pene (−0.19 eV). O_2_ adsorbs physically on P atoms around Au, and the calculated adsorption energy is −0.16 eV, and its dissociation adsorption is slightly less plausible than on pristine Pene considering Au already passivated the lone pair on the P atom beneath it. The further dissociative adsorption of H_2_O and O_2_ onto P atoms of HO-Au_1_-Pene would experience energy barriers of 1.18 and 0.75 eV, respectively, which are also slightly higher than those on pristine Pene [54,55,56,57]. The -OH moiety cannot mediate the dissociation of O_2_, as this requires oxidation of Au(I) that is more demanding than O_2_ dissociation on Pene. Considering these high barriers and the limited adsorption energy of H_2_O and O_2_, degradation of HO-Au_1_-Pene would not take place in the dry air condition that is the common operating condition for NO detection. The spontaneous desorption of H_2_O and O_2_ and reaction of NO make HO-Au_1_-Pene highly selective for NO detection. Considering gold clusters are often poisoned by mercaptanes, the reaction of HO-Au_1_-Pene with H_2_S forming HS-Au_1_-Pene and H_2_O was also investigated. The reaction was found to be 1.03 eV exergonic, suggesting mercaptanes may potentially impact the sensing performance of HO-Au_1_-Pene.

Finally, the sensitivity of HO-Au_1_-Pene for detecting NO was also evaluated. The sensitivity was calculated as $S=\left|exp\left(\frac{E_{g}^{\prime }-E_{g}}{2kT}\right)-1\right|$, where *k* is the Boltzmann constant and $E_{g}^{\prime }-E_{g}$ corresponds to the change of band gap (0.81 eV) in existence of NO. The calculated sensitivity of HO-Au_1_-Pene at 300, 400, and 500 K are all 99.99%. This indicates that HO-Au_1_-Pene is highly sensitive, making it a promising candidate material for detection of NO. The current work demonstrates the working principle of HO-Au_1_-Pene as a sensor for NO detection. It is highly sensitive for NO detection with respect to recent proposed NOx sensors based on phosphorene and other 2D materials (Table 1). The change of band gap in existence of NO (0.81 eV) can in principle be detected as the change of resistance of the fabricated composites. Previously, Au and Pt clusters loaded on carbon nanotubes and black phosphorene, and synthesized black phosphorene, were reported as acceptable for NO_2_ sensing with a similar working principle, and the change of band gap/change of Fermi level due to adsorption/reaction of approaching molecules can be correlated with the change of resistance of the sensor [19,20,46,58].

## 3. Theoretical Methods

All calculations in this study were performed using the PBE functional within the generalized gradient approximation (GGA) using the DSPP potential and DNP basis set as implemented in DMol^3^ [63,64]. Such descriptions of electronic states are of at least double-zeta quality [65], so the calculated energies are expected to exhibit small basis-set superposition errors in principle, together with a reasonable description of weak bonds, within the limits of the theory [66]. The contribution of dispersive interactions to the energetic properties of the reaction species was examined in the benchmark calculations with empirical correction developed by Grimme et al. [67] but was found rather limited and was not considered further. Spin-polarized first-principle-based calculations were performed to investigate the deposition of a Au atom and clusters and the adsorption and reaction concerning NO and NO_2_. Frequency analysis was conducted to ensure that all obtained structures are stable and to derive the partition functions to calculate free energy, in which each transition state structure has only one imaginary frequency in the direction of the reaction. A 4 × 3 supercell of Pene containing 48 atoms was used to mimic the surface of Pene. A 4 × 4 × 1 k-point grid was used to sample the Brillouin zone [68], and the global cutoff radius was 4.50 Å. The convergence criteria for energy and force were set to 1 × 10^−5^ Ha and 2 × 10^−3^ Ha/Å, respectively. With these, the lattice parameters of Pene were calculated to be 4.65 and 3.32 Å, respectively [52,69], and the bulk band gap of Pene was calculated to be 0.87 eV, comparing well with recent theoretical investigations [48,52,53]. The adsorption energy of NO, NO_2_, H_2_O, and O_2_ on pristine Pene were calculated to be −0.21, −0.22, −0.19, and −0.16 eV, respectively, and the calculated energy barriers for H_2_O and O_2_ dissociation were 1.08 and 0.69 eV, respectively. These data, though without empirical correction developed by Grimme et al. [67], vary only within 0.05 eV from those with dispersive interaction included and agree well with reported results, showing the current theoretical approach is already adequate for the investigation [40,54,55,56,57].

## 4. Conclusions

Inspired by the potential significant response of single-site heterogeneous reaction centers, in terms of large band-gap change, etc., to approaching molecules that can be used for sensing applications, we investigated the electronic structure and reaction pathways of Au(I) fragments dispersed on Pene with NO and NO_2_ with extensive first-principle-based calculations. Au_1_-Pene has hybridized Au states in the bulk band gap of Pene and a decreased band gap of 0.14 eV and would aggregate into clusters. Passivation with -OH and -CH_3_ forms thermodynamically plausible HO-Au_1_-Pene and H_3_C-Au_1_-Pene and restores the band gap to that of bulk Pene. HO-Au_1_-Pene and H_3_C-Au_1_-Pene were also examined for detection of NO and NO_2_ that would react with -OH and -CH_3_, and the resulting decrease of band gap back to that of Au_1_-Pene would be measurable. HO-Au_1_-Pene and H_3_C-Au_1_-Pene are highly sensitive to NO and NO_2_, and the calculated theoretical sensitivity are all 99.99%. The calculated energy barrier for the reaction of HO-Au_1_-Pene with NO is 0.36 eV, and the reaction is about thermal neutral, suggesting HO-Au_1_-Pene can be used as NO-sensing material, and the reaction for NO detection would be spontaneous. The reaction of NO_2_ with HO-Au_1_-Pene is endothermic, making the dissociation of product HNO_3_ more plausible, while the barriers for the reaction of CH_3_-Au_1_-Pene with NO and NO_2_ are too high for spontaneous detection. Therefore, HO-Au_1_-Pene is not eligible for NO_2_ sensing and CH_3_-Au_1_-Pene is not eligible for NO and NO_2_ sensing. The reaction of H_2_S with HO-Au_1_-Pene is 1.03 eV exothermic, suggesting HO-Au_1_-Pene should not be used in the existence of mercaptanes. The current work highlights the working principle of HO-Au_1_-Pene as a sensor for NO detection. Such change of band gap/Fermi level in existence of NO (0.81 eV) can in principle be detected as the change of resistance of the fabricated composites, especially when Au fragments are loaded on Pene nanoribbons. Au clusters loaded on carbon nanotubes and Pene were reported as acceptable for gas sensing, and the change of electronic structure upon gas adsorption can be correlated with the change of resistance of the sensor [58]. We expect the findings would pave the way for the design and application of single-site heterogeneous reaction centers for sensing applications.

## Figures and Tables

**Table 1:** Sensing performance of HO-Au_1_-Pene with respect to reported 2D sensors.

Sensor	Analyte	ΔE_g_ (eV)	Sensitivity (%)	Detection Limit	Response Time (s)	Reference
Pene-Au_1_-OH	NO	0.81	99.9			This work
Pene	NO	0.23	26.7			[59]
Pene	NO_2_	0.54	62.8			[59]
V-ε-Pene	NO	0.50	75			[60]
V-ε-Pene	NO_2_	0.30	42.5			[60]
Au doped Pene	NO	0.78				[29]
Ag doped Pene	NO	0.80				[29]
B_3_C	NO	0.73	99.6			[13]
B_3_C	NO_2_	0.04	5.5			[13]
Pene FET	NO_2_		67	100 ppb	75	[19]
MoS_2_	NO_2_		93		~220	[19]
Graphene	NO_2_		13		~240	[19]
Pene	NO_2_		>95		~300	[46]
Pene/AuNPs	NO_2_		1			[46]
Pene/PtNPs	NO_2_		>90			[46]
Pene FET	NO_2_		26	40 ppb	500	[61]
Pene FET	NO_2_	0.28	1600	100 ppb	600	[20]
Pene FET	NO_2_		88	100 ppb	290	[7]
MWCNT/AuNPs	NO_2_		7	0.5 ppm		[58]
MoS_2_/PtNPs	NO_2_		2	0.025 ppb		[62]

## Data Availability

The original contributions presented in this study are included in the article/Supplementary Material. Further inquiries can be directed to the corresponding author.

## Supplementary Materials

The following supporting information can be downloaded at https://www.mdpi.com/article/10.3390/molecules30153085/s1, cartesian coordinates of Au fragments on Pene in xyz format.
