# Supplementary material for: Phosphorene-Supported Au(I) Fragments for Highly Sensitive Detection of NO
Source: Molecules. 2025 Jul 23;30(15):3085. doi: 10.3390/molecules30153085 (PMC12348119; doi:10.3390/molecules30153085)
Supplement: Supplementary file 1 [file molecules-30-03085-s001.zip › molecules-3725902-supplementary.pdf]

Supporting Information

Phosphorene-Supported Au(I) Fragments for Highly Sensitive Detection of NO

Huimin Guo*, Yuhan Liu, Xin Liu

School of Chemistry, State Key Laboratory of Fine Chemicals, Frontier Science Center for Smart Materials, Dalian Key Laboratory of Intelligent Chemistry, Dalian University of Technology, Dalian 116024, China

*Corresponding author, E-mail: guohm@dlut.edu.cn(H.G.)

FigureS1a

P1	1.924518	1.655164	5.587556
P2	4.263861	0.001507	3.481807
P3	0.428898	-0.000935	5.605135
P4	2.740955	1.639764	3.453676
P5	6.560274	1.669644	5.648213
P6	8.896177	0.012514	3.513207
P7	5.076620	-0.001820	5.624466
P8	7.402726	1.680867	3.520322
P9	11.241140	1.666253	5.616794
P10	13.545596	0.003633	3.474654
P11	9.742993	0.010016	5.635274
P12	12.052289	1.667891	3.477505
P13	1.924778	4.979584	5.601276
P14	4.236328	3.310541	3.504084
P15	0.432511	3.316636	5.597257
P16	2.742681	4.973462	3.470047
P17	6.441926	4.977052	5.605270
P18	8.900219	3.342838	3.524384
P19	4.980433	3.265145	5.659697
P20	7.405273	4.998000	3.460500
P21	11.312283	4.958030	5.625094
P22	13.559599	3.319198	3.464390
P23	9.748456	3.340814	5.657667
P24	12.070639	4.992230	3.473245
P25	1.929463	8.309745	5.597535
P26	4.243165	6.634144	3.477865
P27	0.436981	6.646823	5.601142
P28	2.753818	8.307215	3.465442
P29	6.566310	8.286523	5.656742
P30	8.904755	6.626894	3.462187
P31	4.999058	6.670946	5.630124
P32	7.411276	8.283691	3.523327
P33	11.331542	8.360396	5.659063
P34	13.570122	6.653608	3.469396
P35	9.864596	6.647708	5.606639
P36	12.076026	8.316124	3.504546
P37	1.933964	11.627150	5.605658
P38	4.261731	9.958132	3.478957
P39	0.437695	9.971418	5.587309
P40	2.769051	11.622414	3.475895
P41	6.571419	11.616647	5.635993
P42	8.909545	9.945040	3.519293
P43	5.073892	9.959658	5.618521
P44	7.417123	11.614141	3.513425
P45	11.236812	11.627903	5.622742
P46	13.572564	9.986472	3.452909

P47	9.753732	9.956409	5.646885
P48	12.050242	11.625087	3.480260
Au	8.165899	5.776584	7.077651

FigureS1b

P1	1.829940	1.701858	5.595103
P2	4.124345	0.012271	3.458901
P3	0.339748	0.040291	5.592538
P4	2.652029	1.698872	3.463567
P5	6.373717	1.654289	5.618148
P6	8.803254	0.058456	3.610317
P7	4.871781	0.002240	5.599323
P8	7.310075	1.701219	3.504301
P9	11.244799	1.734834	5.622031
P10	13.451248	0.022502	3.469366
P11	9.705388	0.096480	5.728410
P12	11.969143	1.703313	3.462972
P13	1.822037	5.022171	5.594975
P14	4.154773	3.361482	3.482960
P15	0.331775	3.360524	5.592391
P16	2.663637	5.033841	3.473105
P17	6.420739	4.978527	5.752928
P18	8.799520	3.360250	3.515730
P19	4.887285	3.327776	5.644803
P20	7.308329	4.999970	3.628162
P21	11.234696	5.054097	5.602363
P22	13.468339	3.369999	3.457024
P23	9.740414	3.389702	5.631676
P24	11.984251	5.047059	3.460376
P25	1.828840	8.343429	5.611450
P26	4.181013	6.671346	3.547219
P27	0.334158	6.687365	5.584974
P28	2.680654	8.329818	3.490481
P29	6.449754	8.342560	5.706707
P30	8.795352	6.667510	3.586054
P31	4.936796	6.694420	5.712893
P32	7.306259	8.334654	3.587548
P33	11.155840	8.340519	5.661994
P34	13.477244	6.720948	3.449169
P35	9.681920	6.665816	5.687903
P36	11.939423	8.345593	3.509418
P37	1.826166	11.654448	5.581186
P38	4.163799	9.994880	3.498642
P39	0.330667	9.999133	5.615844
P40	2.627063	11.620958	3.443695
P41	6.423472	11.670402	5.684869
P42	8.801320	10.001749	3.586563
P43	4.948835	9.997669	5.651434
P44	7.314991	11.670648	3.584046

P45	11.163431	11.645575	5.722898
P46	13.418981	10.013077	3.498731
P47	9.651262	9.994845	5.708263
P48	11.923474	11.674029	3.554611
Au1	8.483162	1.301524	7.434768
Au2	7.570557	3.785134	7.539618

FigureS1c

P1	1.870697	1.670137	5.501475
P2	0.382340	0.006149	5.510482
P3	2.677995	1.664852	3.369530
P4	6.398768	1.651238	5.499389
P5	8.863082	0.027997	3.513827
P6	7.364051	1.664861	3.408919
P7	11.286623	1.678949	5.536198
P8	13.524602	-0.019893	3.378200
P9	9.725860	0.079568	5.648525
P10	12.024991	1.648988	3.387446
P11	1.869136	4.992026	5.497835
P12	4.175208	3.335020	3.418352
P13	0.375926	3.331377	5.518429
P14	2.684033	5.012501	3.368789
P15	6.406885	4.974692	5.522539
P16	8.844560	3.336749	3.456631
P17	4.880847	3.329306	5.580159
P18	7.363014	4.994680	3.432595
P19	11.254279	5.009442	5.527856
P20	13.506356	3.332063	3.388782
P21	9.758444	3.345780	5.562513
P22	12.018438	5.007395	3.382357
P23	1.879154	8.310684	5.524012
P24	4.208786	6.647542	3.442758
P25	0.381409	6.654970	5.517156
P26	2.716145	8.315668	3.397598
P27	6.510499	8.315759	5.602158
P28	8.860096	6.650416	3.517606
P29	4.970095	6.679420	5.607192
P30	7.367694	8.313295	3.484544
P31	11.209481	8.321730	5.575167
P32	13.513110	6.675940	3.384423
P33	9.719078	6.633034	5.638421
P34	11.994962	8.317871	3.424694
P35	1.876989	11.626554	5.516873
P36	4.221744	9.967367	3.419604
P37	0.383984	9.967871	5.528744
P38	2.699472	11.607080	3.385516
P39	6.493506	11.640088	5.599111
P40	8.861798	9.981460	3.494597

P41	5.007837	9.970569	5.569595
P42	7.369708	11.644996	3.489155
P43	11.229598	11.630935	5.611505
P44	13.505070	9.966395	3.400402
P45	9.714101	9.983753	5.615247
P46	12.000438	11.628428	3.452845
P47	4.182478	13.284349	3.394606
P48	4.937412	13.259615	5.548625
Au1	8.165493	1.396486	7.101008
Au2	7.926585	4.422432	7.214765
Au3	8.753292	2.911310	9.267302

FigureS2a

P1	1.743170	1.664455	5.634600
P2	4.083223	0.007125	3.529011
P3	0.253709	0.002264	5.637231
P4	2.570424	1.654756	3.503632
P5	6.377174	1.666348	5.704280
P6	8.721700	0.010563	3.571068
P7	4.883382	0.003970	5.673720
P8	7.231789	1.676787	3.581209
P9	11.092907	1.655569	5.662424
P10	13.382116	0.002520	3.503606
P11	9.583462	0.007872	5.689672
P12	11.879990	1.659764	3.520932
P13	1.736402	4.986111	5.647593
P14	4.059599	3.323283	3.520916
P15	0.246382	3.321476	5.647967
P16	2.564895	4.988145	3.519051
P17	6.265861	4.985220	5.602955
P18	8.739115	3.326256	3.613359
P19	4.828164	3.283968	5.672976
P20	7.254570	4.985833	3.541616
P21	11.088208	4.987390	5.711870
P22	13.358265	3.333987	3.521055
P23	9.589444	3.319415	5.737313
P24	11.854089	4.988002	3.558110
P25	1.742793	8.308235	5.638418
P26	4.066498	6.646656	3.533484
P27	0.246192	6.650738	5.647623
P28	2.578123	8.314753	3.510276
P29	6.383903	8.311081	5.707338
P30	8.739120	6.647528	3.617629
P31	4.838128	6.689656	5.688383
P32	7.233255	8.296706	3.582874
P33	11.095946	8.317598	5.655432
P34	13.360925	6.639570	3.519472
P35	9.590269	6.657295	5.741391

P36	11.882804	8.314331	3.515493
P37	1.752317	11.626400	5.643032
P38	4.086272	9.965243	3.533879
P39	0.253708	9.971081	5.636393
P40	2.590229	11.624832	3.515282
P41	6.384012	11.629067	5.692954
P42	8.721988	9.963634	3.571526
P43	4.886080	9.971649	5.678096
P44	7.228533	11.627161	3.568397
P45	11.075325	11.627110	5.656233
P46	13.384710	9.972503	3.501888
P47	9.586576	9.964351	5.688904
P48	11.882116	11.626775	3.513419
Au	7.665867	4.934281	7.375439
O	8.906935	4.831507	8.959051
H	8.382490	5.097293	9.736572

FigureS2b

P1	1.951214	1.632854	5.553158
P2	4.284800	-0.025076	3.439520
P3	0.457281	-0.024750	5.561477
P4	2.776626	1.624690	3.411796
P5	6.568649	1.627161	5.641856
P6	8.923344	-0.023092	3.509175
P7	5.068552	-0.026574	5.604504
P8	7.428616	1.639085	3.510934
P9	11.294482	1.626459	5.593562
P10	13.579639	-0.026362	3.419698
P11	9.789680	-0.023267	5.634942
P12	12.080252	1.630660	3.435955
P13	1.936285	4.955151	5.557919
P14	4.266949	3.291625	3.436433
P15	0.453387	3.287506	5.563292
P16	2.765742	4.950849	3.421496
P17	6.457991	4.932135	5.542088
P18	8.933394	3.290898	3.551852
P19	5.016162	3.239706	5.607165
P20	7.452662	4.955198	3.481831
P21	11.307068	4.945345	5.616810
P22	13.572980	3.292758	3.424254
P23	9.799576	3.291538	5.675452
P24	12.065582	4.946076	3.455052
P25	1.935060	8.277265	5.554640
P26	4.260570	6.615145	3.449614
P27	0.444632	6.615887	5.568549
P28	2.766113	8.280193	3.419103
P29	6.467353	8.297068	5.523151
P30	8.940515	6.612866	3.573445

P31	4.992416	6.620127	5.628608
P32	7.458509	8.271300	3.452759
P33	11.313189	8.286339	5.621003
P34	13.550869	6.615470	3.436070
P35	9.811555	6.622773	5.693350
P36	12.062616	8.283681	3.457821
P37	1.948882	11.595918	5.552940
P38	4.266964	9.938769	3.436311
P39	0.451063	9.942572	5.559609
P40	2.778245	11.604768	3.412748
P41	6.572354	11.603403	5.636423
P42	8.937317	9.937502	3.545448
P43	5.026136	9.985881	5.606141
P44	7.433681	11.590757	3.506873
P45	11.287588	11.601473	5.599326
P46	13.567359	9.938666	3.421841
P47	9.794029	9.928759	5.673953
P48	12.075924	11.599984	3.439849
Au1	7.793247	5.016803	7.373018
O1	8.769861	5.271694	9.146000
Au2	7.875339	8.215961	7.309118
O2	8.903811	8.078725	9.033457
H1	9.609407	4.778503	9.110821
H2	8.923425	7.092849	9.200293

FigureS2c

P1	1.888166	1.615487	5.522216
P2	4.223235	-0.049593	3.420630
P3	0.397460	-0.041480	5.543941
P4	2.697822	1.589203	3.374741
P5	6.464902	1.614458	5.659150
P6	8.864628	-0.035806	3.546660
P7	4.986169	-0.056581	5.590544
P8	7.376459	1.629469	3.550238
P9	11.234671	1.618796	5.646940
P10	13.492220	-0.019684	3.413909
P11	9.735885	-0.040808	5.670072
P12	11.984084	1.629487	3.473581
P13	1.884348	4.935806	5.544775
P14	4.187939	3.259646	3.381890
P15	0.397185	3.274334	5.549157
P16	2.716691	4.949139	3.407676
P17	6.347988	4.876296	5.588820
P18	8.883024	3.281724	3.601333
P19	4.892813	3.206972	5.551147
P20	7.396195	4.933046	3.550773
P21	11.284738	4.909832	5.687172
P22	13.476976	3.288402	3.426338

P23	9.731876	3.284685	5.730091
P24	11.989489	4.953641	3.490216
P25	1.893240	8.266342	5.544044
P26	4.240334	6.585910	3.473344
P27	0.403684	6.606426	5.548845
P28	2.751641	8.250959	3.416750
P29	6.491236	8.267199	5.715820
P30	8.852412	6.612661	3.592635
P31	4.958769	6.623950	5.665401
P32	7.365739	8.262042	3.596450
P33	11.347786	8.339991	5.559292
P34	13.512682	6.589958	3.415299
P35	9.901697	6.664103	5.630277
P36	12.044624	8.281993	3.387847
P37	1.893741	11.582431	5.537202
P38	4.243867	9.912030	3.456537
P39	0.401983	9.926143	5.525106
P40	2.737691	11.563713	3.402714
P41	6.494634	11.579834	5.662077
P42	8.870094	9.915053	3.554520
P43	4.988488	9.926152	5.628905
P44	7.377023	11.576255	3.542772
P45	11.248924	11.599553	5.602336
P46	13.535659	9.952794	3.379504
P47	9.771318	9.928027	5.668624
P48	12.011101	11.591042	3.432179
Au1	7.444951	4.524573	7.547371
O1	8.130511	4.182547	9.411966
Au2	8.884354	7.006131	7.634598
O2	8.286022	7.199713	9.565936
H1	8.129121	5.092841	9.796727
H2	7.433991	7.674501	9.560773

FigureS2d

P1	1.962632	1.635439	5.557856
P2	2.790902	1.638230	3.419596
P3	6.487858	1.635801	5.499936
P4	7.474039	1.634820	3.426522
P5	11.305309	1.631594	5.621208
P6	12.080854	1.632235	3.456028
P7	1.961963	4.954821	5.544982
P8	4.286396	3.299825	3.429862
P9	0.471279	3.295610	5.562312
P10	2.790299	4.960309	3.405082
P11	6.600294	4.951676	5.629894
P12	8.954867	3.297767	3.525875
P13	5.051086	3.333289	5.596891
P14	7.455543	4.955586	3.498875
P15	11.319411	4.952398	5.582463

P16	13.585067	3.284032	3.424362
P17	9.811159	3.295201	5.654237
P18	12.099740	4.952539	3.429230
P19	1.962630	8.275646	5.557581
P20	4.289991	6.617003	3.438040
P21	0.471525	6.614014	5.561907
P22	2.789822	8.273234	3.419395
P23	6.487441	8.280059	5.507178
P24	8.954789	6.610936	3.532865
P25	5.060359	6.577499	5.609819
P26	7.473202	8.274699	3.435038
P27	11.307817	8.273554	5.619281
P28	13.586550	6.620375	3.423606
P29	9.811580	6.609586	5.661864
P30	12.082294	8.271891	3.454885
P31	1.963880	11.595446	5.540824
P32	4.281776	9.939593	3.420783
P33	0.471242	9.936762	5.560180
P34	2.784209	11.601188	3.399170
P35	6.594000	11.589751	5.626564
P36	8.955277	9.936935	3.524083
P37	5.041776	9.973492	5.585548
P38	7.452655	11.592768	3.496501
P39	11.326198	11.592501	5.573978
P40	13.590025	9.921867	3.421714
P41	9.815270	9.938964	5.650488
P42	12.104653	11.592667	3.422085
P43	4.287163	13.257467	3.428457
P44	0.471632	13.253765	5.560198
P45	8.951763	13.249613	3.526167
P46	5.055584	13.217707	5.598608
P47	13.588831	13.263802	3.421668
P48	9.812186	13.246174	5.652948
Au1	7.898919	1.640778	7.275462
O1	8.968280	1.722677	8.984885
Au2	7.881670	8.371004	7.287479
O2	8.998344	8.567976	8.955838
H1	8.858164	0.850993	9.411420
H2	8.621498	7.947114	9.607383

FigureS2e

P1	1.966501	1.664429	5.535751
P2	4.311327	0.009957	3.450807
P3	0.478055	0.000615	5.550717
P4	2.784465	1.643159	3.394859
P5	6.485311	1.698106	5.508188
P6	8.972497	0.001010	3.536977
P7	5.085083	-0.027685	5.626620

P8	7.493084	1.668333	3.449965
P9	11.331456	1.659032	5.609804
P10	13.582477	-0.005099	3.416715
P11	9.819077	0.010200	5.669921
P12	12.090899	1.658553	3.441027
P13	1.980662	4.983078	5.526054
P14	4.267406	3.326121	3.370277
P15	0.480707	3.329535	5.531177
P16	2.794841	5.011986	3.383815
P17	6.568310	4.978194	5.641592
P18	8.970962	3.333119	3.530004
P19	5.003265	3.362931	5.527282
P20	7.453049	4.974735	3.524397
P21	11.396777	4.951585	5.543691
P22	13.608422	3.302752	3.392698
P23	9.842486	3.328894	5.652981
P24	12.133486	4.986723	3.384156
P25	1.983160	8.310413	5.551033
P26	4.318116	6.650122	3.437714
P27	0.495070	6.647563	5.538459
P28	2.834045	8.318578	3.418208
P29	6.598517	8.294408	5.662782
P30	8.921885	6.643387	3.444283
P31	5.085079	6.648569	5.607828
P32	7.445704	8.310860	3.530014
P33	11.332220	8.340408	5.617423
P34	13.621112	6.663941	3.399278
P35	9.921439	6.619536	5.510128
P36	12.103163	8.304250	3.444172
P37	1.975177	11.626101	5.553335
P38	4.334527	9.970636	3.455353
P39	0.486809	9.964992	5.551616
P40	2.829708	11.620465	3.420138
P41	6.621335	11.620942	5.641840
P42	8.950804	9.961423	3.504678
P43	5.112664	9.968846	5.618474
P44	7.465597	11.632702	3.506788
P45	11.308679	11.620635	5.610467
P46	13.589567	9.970973	3.416067
P47	9.798991	9.971148	5.638562
P48	12.083634	11.620389	3.448134
Au1	7.713131	2.160236	7.355158
O1	8.532016	2.800873	9.108173
Au2	8.663803	6.157961	7.346608
O2	7.878904	5.564832	9.096744
H1	7.992913	2.393011	9.811532
H2	8.120254	4.593758	9.148044

FigureS3a

P1	1.932417	1.624062	5.594145
P2	2.753851	1.616898	3.449050
P3	6.586047	1.620420	5.595791
P4	7.419066	1.630261	3.453996
P5	11.241555	1.617635	5.601332
P6	12.070638	1.618526	3.459929
P7	1.928034	4.944718	5.601226
P8	4.241540	3.282197	3.451048
P9	0.438401	3.282107	5.608877
P10	2.750242	4.945241	3.459595
P11	6.496232	4.942451	5.495154
P12	8.918040	3.285038	3.492027
P13	5.054680	3.251686	5.600685
P14	7.438575	4.944758	3.396124
P15	11.232885	4.946114	5.636766
P16	13.545738	3.290665	3.472068
P17	9.731965	3.283296	5.643238
P18	12.045009	4.945160	3.488442
P19	1.932545	8.265222	5.595375
P20	4.244649	6.605413	3.455276
P21	0.438315	6.607439	5.608723
P22	2.757211	8.270607	3.451308
P23	6.589463	8.269631	5.596563
P24	8.917803	6.605323	3.489389
P25	5.059409	6.636605	5.605940
P26	7.418710	8.260077	3.453474
P27	11.243274	8.273714	5.598198
P28	13.546413	6.599202	3.471545
P29	9.733368	6.609857	5.639508
P30	12.072333	8.272414	3.457406
P31	1.941551	11.584683	5.601213
P32	4.257749	9.924134	3.452989
P33	0.445378	9.928349	5.601297
P34	2.765614	11.583846	3.455879
P35	6.584921	11.585224	5.594056
P36	8.909098	9.924372	3.457061
P37	5.091998	9.926669	5.597537
P38	7.416139	11.585276	3.450806
P39	11.234316	11.585176	5.593886
P40	13.572204	9.928886	3.455345
P41	9.745165	9.925423	5.598116
P42	12.076244	11.585107	3.451352
P43	4.256289	13.244699	3.451291
P44	0.445226	13.241014	5.601925
P45	8.909257	13.246118	3.457784
P46	5.090390	13.242306	5.596022
P47	13.570731	13.242241	3.456456

P48	9.744626	13.244663	5.599018
Au1	7.975843	4.941712	7.341751
C1	9.015980	5.011052	9.153328
H1	8.708100	5.923843	9.681857
H2	8.741610	4.124474	9.741224
H3	10.100763	5.025090	8.984814

FigureS3b

P1	1.968546	1.053300	5.571230
P2	2.794227	1.046995	3.428709
P3	6.615289	1.047646	5.595833
P4	7.445167	1.058057	3.453325
P5	11.292799	1.044451	5.565756
P6	12.116277	1.044659	3.420795
P7	1.953100	4.372046	5.572560
P8	4.283096	2.711888	3.439693
P9	0.468539	2.706173	5.577633
P10	2.781832	4.368662	3.434355
P11	6.522478	4.367428	5.506199
P12	8.943072	2.714949	3.492317
P13	5.079658	2.676439	5.596898
P14	7.466483	4.378406	3.407425
P15	11.286308	4.362944	5.613831
P16	13.592636	2.716437	3.435357
P17	9.781294	2.705410	5.634225
P18	12.078748	4.363077	3.464917
P19	1.952910	7.693364	5.572413
P20	4.272855	6.033368	3.455466
P21	0.461282	6.032654	5.586991
P22	2.780630	7.696819	3.433693
P23	6.522929	7.698936	5.508051
P24	8.953988	6.032936	3.519001
P25	5.043952	6.033086	5.622577
P26	7.466308	7.687277	3.409470
P27	11.286840	7.702566	5.612336
P28	13.562706	6.032490	3.454788
P29	9.772722	6.032954	5.669510
P30	12.079501	7.702540	3.463316
P31	1.968238	11.012451	5.571435
P32	4.281824	9.353554	3.439249
P33	0.468567	9.359348	5.578027
P34	2.793506	11.018960	3.428710
P35	6.615071	11.017095	5.594551
P36	8.943259	9.350377	3.490241
P37	5.078785	9.389508	5.596135
P38	7.445181	11.007833	3.452219
P39	11.293046	11.021270	5.565852
P40	13.593236	9.349450	3.435519

P41	9.782284	9.360024	5.632016
P42	12.116750	11.021192	3.420962
P43	4.294604	12.672738	3.438807
P44	0.478333	12.672968	5.569953
P45	8.937109	12.673034	3.454045
P46	5.115440	12.672430	5.590299
P47	13.622753	12.673099	3.419084
P48	9.797228	12.672768	5.584593
Au1	7.998650	4.290408	7.355087
C1	9.048290	4.153407	9.157290
H1	8.522744	4.769413	9.898871
H2	9.054855	3.100415	9.471099
H3	10.077922	4.512795	9.034152
Au2	7.999725	7.778752	7.356124
C2	9.049412	7.908967	9.158797
H4	9.343913	8.953510	9.327558
H5	8.369250	7.574762	9.954102
H6	9.934719	7.261458	9.130562

FigureS3c

P1	2.025360	1.067515	5.564749
P2	2.831549	1.055736	3.415960
P3	6.657797	1.061358	5.588988
P4	7.501765	1.071618	3.450565
P5	11.347520	1.063504	5.593169
P6	12.157831	1.068768	3.446040
P7	2.023545	4.386331	5.584125
P8	4.326177	2.719410	3.407602
P9	0.530595	2.726258	5.582147
P10	2.855875	4.402897	3.447393
P11	6.585263	4.342446	5.524374
P12	9.004785	2.729395	3.460280
P13	5.111016	2.680060	5.556804
P14	7.518241	4.379980	3.424735
P15	11.345539	4.369514	5.654478
P16	13.637315	2.739323	3.448974
P17	9.824813	2.719489	5.608124
P18	12.138133	4.394751	3.489523
P19	2.029620	7.713968	5.580897
P20	4.373675	6.044696	3.493334
P21	0.536806	6.053472	5.584441
P22	2.875027	7.700613	3.448797
P23	6.685708	7.720620	5.609966
P24	8.990760	6.061194	3.424086
P25	5.164815	6.070654	5.658529
P26	7.503496	7.710577	3.461338
P27	11.399336	7.760264	5.557399
P28	13.655135	6.037394	3.447715

P29	9.925780	6.097731	5.522636
P30	12.184080	7.720613	3.408391
P31	2.029646	11.031340	5.569911
P32	4.354033	9.371679	3.448487
P33	0.534577	9.372742	5.564707
P34	2.843485	11.024358	3.426089
P35	6.669790	11.029282	5.587217
P36	9.006988	9.367905	3.451642
P37	5.163248	9.377152	5.595244
P38	7.508575	11.027680	3.449897
P39	11.345346	11.042795	5.565354
P40	13.678892	9.384494	3.416314
P41	9.852273	9.378671	5.589695
P42	12.170515	11.036296	3.419083
P43	4.339805	12.683963	3.420816
P44	0.530817	12.688636	5.570479
P45	9.001692	12.693046	3.449578
P46	5.164579	12.677483	5.567442
P47	13.667372	12.695454	3.426285
P48	9.841106	12.691785	5.586531
Au1	7.968005	3.799962	7.413221
C1	8.898184	3.229300	9.198756
H1	8.288111	3.614584	10.026543
H2	8.938889	2.132208	9.226247
H3	9.909941	3.651850	9.242729
Au2	8.548491	6.629334	7.419020
C2	7.624811	7.215268	9.200615
H4	7.690375	8.309524	9.269370
H5	6.575253	6.894669	9.204561
H6	8.170056	6.740529	10.026701

FigureS3d

P1	1.959092	1.038857	5.578629
P2	2.777208	1.038894	3.434775
P3	6.621995	1.038649	5.590961
P4	7.447358	1.038832	3.448958
P5	11.291217	1.039272	5.579779
P6	12.109172	1.038931	3.441419
P7	1.963593	4.359138	5.592449
P8	4.274923	2.697556	3.443904
P9	0.468924	2.699850	5.594801
P10	2.782307	4.360072	3.450527
P11	6.526784	4.359291	5.489650
P12	8.945467	2.698377	3.482114
P13	5.084667	2.668805	5.596454
P14	7.464440	4.358398	3.387833
P15	11.267939	4.358392	5.616383
P16	13.592739	2.709516	3.453397

P17	9.772253	2.693869	5.627786
P18	12.085347	4.358693	3.467082
P19	1.959019	7.678914	5.579880
P20	4.276090	6.021277	3.446324
P21	0.468833	6.018273	5.597054
P22	2.777981	7.678975	3.435458
P23	6.623598	7.678797	5.592968
P24	8.945993	6.019406	3.482317
P25	5.085656	6.049872	5.600084
P26	7.447677	7.678831	3.450104
P27	11.281496	7.678359	5.587164
P28	13.588923	6.010906	3.456255
P29	9.768831	6.020561	5.629175
P30	12.104089	7.678799	3.446770
P31	1.963486	10.998653	5.592327
P32	4.275804	9.336644	3.446201
P33	0.468747	9.339464	5.597141
P34	2.781983	10.997665	3.450461
P35	6.527033	10.998545	5.489525
P36	8.946315	9.338354	3.482530
P37	5.085971	9.308058	5.600484
P38	7.464974	10.999064	3.387248
P39	11.268197	10.999248	5.616496
P40	13.588490	9.346825	3.456412
P41	9.768188	9.337052	5.629474
P42	12.084477	10.999019	3.467811
P43	4.274713	12.660138	3.443571
P44	0.468792	12.657932	5.594924
P45	8.945846	12.659331	3.482120
P46	5.085062	12.688612	5.596264
P47	13.592314	12.648242	3.453548
P48	9.771673	12.663779	5.628250
Au1	8.005293	4.315876	7.334442
C1	9.045801	4.224849	9.143737
H1	8.515431	4.853007	9.872672
H2	9.050560	3.178683	9.479666
H3	10.076159	4.582814	9.021675
Au2	8.005289	11.042837	7.334628
C2	9.041972	11.128840	9.146227
H4	9.051000	12.175024	9.482401
H5	8.506748	10.501654	9.872754
H6	10.070819	10.765570	9.025003

FigureS3e

P1	2.039338	1.076123	5.618267
P2	2.849886	1.071490	3.469944
P3	6.693946	1.068265	5.615078
P4	7.527602	1.077499	3.474751

P5	11.357636	1.064290	5.613386
P6	12.187744	1.062987	3.472475
P7	2.035821	4.389356	5.624480
P8	4.343366	2.731907	3.468415
P9	0.540264	2.730503	5.633046
P10	2.845037	4.388752	3.478723
P11	6.597921	4.397185	5.517375
P12	9.027497	2.733827	3.504677
P13	5.165028	2.699880	5.619330
P14	7.545812	4.393954	3.419886
P15	11.344070	4.389975	5.656600
P16	13.659078	2.738973	3.493179
P17	9.843325	2.725701	5.655590
P18	12.155799	4.392456	3.507109
P19	2.047177	7.710836	5.625011
P20	4.347073	6.046993	3.474882
P21	0.549842	6.054944	5.625119
P22	2.877506	7.726385	3.485661
P23	6.710012	7.715267	5.662901
P24	9.025694	6.056437	3.511302
P25	5.152977	6.081447	5.621468
P26	7.519835	7.708619	3.509638
P27	11.392471	7.685906	5.621891
P28	13.668903	6.038799	3.486115
P29	9.836600	6.051542	5.664218
P30	12.199436	7.718438	3.475433
P31	2.057159	11.034599	5.632689
P32	4.390460	9.372685	3.504979
P33	0.560954	9.376343	5.623586
P34	2.888086	11.026752	3.492646
P35	6.703610	11.037846	5.654647
P36	8.998983	9.371330	3.415816
P37	5.201278	9.375246	5.654603
P38	7.518509	11.031670	3.503471
P39	11.380365	11.065475	5.618071
P40	13.701653	9.376677	3.478343
P41	9.946589	9.369441	5.513873
P42	12.203631	11.034348	3.468033
P43	4.360181	12.702065	3.471743
P44	0.558114	12.688723	5.617994
P45	9.017362	12.689202	3.474207
P46	5.189237	12.700658	5.612874
P47	13.698437	12.694007	3.469683
P48	9.852042	12.697181	5.614654
Au1	8.113827	4.372253	7.335786
C1	9.231017	4.293545	9.101970
H1	8.739902	4.936033	9.845610
H2	9.240922	3.250998	9.448729

H3	10.259409	4.638477	8.932354
Au2	8.437171	9.391498	7.339109
C2	7.343317	9.447564	9.122144
H4	7.188050	10.498574	9.401922
H5	6.373986	8.947524	8.996984
H6	7.934095	8.934190	9.892569

FigureS4a

P1	1.473369	1.691902	5.513700
P2	3.816246	0.033815	3.403906
P3	-0.017115	0.030047	5.509793
P4	2.311889	1.687076	3.383414
P5	6.134238	1.681672	5.554909
P6	8.453678	0.034456	3.414480
P7	4.626469	0.028846	5.546967
P8	6.963870	1.700213	3.414976
P9	10.816194	1.680120	5.516334
P10	13.117616	0.030262	3.370134
P11	9.307143	0.034456	5.540376
P12	11.615078	1.687468	3.375542
P13	1.467567	5.012817	5.520913
P14	3.802533	3.351827	3.406069
P15	-0.023495	3.349175	5.521805
P16	2.302389	5.010348	3.391740
P17	6.009775	5.015454	5.451674
P18	8.467411	3.351929	3.458329
P19	4.587892	3.305134	5.560877
P20	6.977373	5.009161	3.380070
P21	10.804458	5.013071	5.573196
P22	13.093014	3.362067	3.390189
P23	9.311115	3.340956	5.589604
P24	11.585196	5.013771	3.421857
P25	1.473886	8.334753	5.511566
P26	3.797664	6.673334	3.396799
P27	-0.022728	6.677321	5.520050
P28	2.308719	8.340847	3.380658
P29	6.122378	8.339736	5.559030
P30	8.459477	6.673012	3.449869
P31	4.578475	6.715592	5.550041
P32	6.956397	8.327089	3.427137
P33	10.813022	8.345178	5.515715
P34	13.093210	6.664873	3.389472
P35	9.305243	6.680086	5.576650
P36	11.616533	8.340144	3.377544
P37	1.481959	11.654139	5.516617
P38	3.814872	9.993266	3.399577
P39	-0.016082	9.997269	5.508512
P40	2.320197	11.653863	3.386124

P41	6.126636	11.653301	5.549552
P42	8.449357	9.990590	3.414030
P43	4.624173	9.999532	5.543450
P44	6.956343	11.654555	3.415805
P45	10.798538	11.653610	5.512801
P46	13.119643	9.997995	3.369878
P47	9.307036	9.993083	5.536230
P48	11.618404	11.653506	3.371208
Au	7.435350	5.080054	7.228912
O1	7.281361	2.410030	9.258045
N1	6.455938	3.004505	8.700153
O2	8.591960	5.116678	8.891612
H1	8.642680	6.050724	9.166148

FigureS4b

P1	1.465243	1.711301	5.516907
P2	3.805533	0.055150	3.402844
P3	-0.025892	0.048931	5.520154
P4	2.300895	1.707873	3.387452
P5	6.122875	1.708921	5.555763
P6	8.447180	0.054772	3.423930
P7	4.617580	0.052995	5.543253
P8	6.955846	1.721564	3.426578
P9	10.800557	1.700989	5.548958
P10	13.099037	0.047335	3.386896
P11	9.286180	0.055790	5.551829
P12	11.595982	1.707220	3.408204
P13	1.459187	5.031693	5.516635
P14	3.788891	3.378965	3.400611
P15	-0.032082	3.368890	5.520061
P16	2.279196	5.031392	3.385546
P17	6.003213	5.053485	5.420937
P18	8.458856	3.376062	3.451209
P19	4.583602	3.344770	5.550352
P20	6.976276	5.029293	3.330984
P21	10.828061	5.033988	5.529452
P22	13.096180	3.362947	3.386770
P23	9.303696	3.399712	5.590846
P24	11.600693	5.026388	3.382148
P25	1.463809	8.353345	5.506066
P26	3.769541	6.699072	3.388838
P27	-0.032630	6.694586	5.520451
P28	2.277836	8.370116	3.371030
P29	6.103647	8.347251	5.551207
P30	8.462226	6.689039	3.431159
P31	4.545216	6.735747	5.531190
P32	6.953883	8.342179	3.425201
P33	10.797922	8.361033	5.533817

P34	13.094111	6.692754	3.388252
P35	9.308223	6.672782	5.565139
P36	11.600150	8.356088	3.392958
P37	1.472019	11.671759	5.524097
P38	3.796993	10.011793	3.396656
P39	-0.027306	10.015044	5.520870
P40	2.306851	11.676114	3.393688
P41	6.110945	11.670407	5.550438
P42	8.443658	10.008986	3.423294
P43	4.612189	10.012795	5.538149
P44	6.949472	11.672580	3.421295
P45	10.779864	11.673900	5.532848
P46	13.099064	10.017516	3.387485
P47	9.289593	10.009235	5.548022
P48	11.597567	11.672603	3.393226
Au1	7.572914	5.072186	7.131239
O1	8.792557	3.812675	9.720803
N1	8.028419	4.630359	9.340071
O2	8.867291	6.145209	9.051917
H1	8.145537	6.783201	9.218318

FigureS4c

P1	1.489461	1.702933	5.500483
P2	3.823405	0.042762	3.384372
P3	0.000166	0.040332	5.521649
P4	2.302038	1.684152	3.361751
P5	6.125972	1.701003	5.545356
P6	8.474417	0.045103	3.428975
P7	4.629355	0.040636	5.530224
P8	6.977577	1.707384	3.422969
P9	10.805904	1.698147	5.562301
P10	13.114588	0.040653	3.390617
P11	9.301508	0.045737	5.563000
P12	11.608796	1.698225	3.418530
P13	1.484796	5.023573	5.507592
P14	3.790771	3.356012	3.373832
P15	-0.007843	3.361403	5.521460
P16	2.294707	5.021456	3.370736
P17	6.004137	5.016784	5.391739
P18	8.477790	3.365429	3.462329
P19	4.569450	3.319440	5.524669
P20	6.994040	5.024284	3.330322
P21	10.834165	5.027011	5.554762
P22	13.100213	3.360636	3.395500
P23	9.302557	3.394516	5.598004
P24	11.609093	5.027698	3.404536
P25	1.489645	8.344650	5.502575
P26	3.794031	6.684765	3.375769

P27	-0.007953	6.686472	5.519673
P28	2.306328	8.357448	3.365530
P29	6.123545	8.340875	5.549352
P30	8.477166	6.682017	3.459000
P31	4.572296	6.715418	5.527191
P32	6.978361	8.339298	3.427594
P33	10.826492	8.355998	5.543340
P34	13.108395	6.687652	3.390786
P35	9.313536	6.666509	5.590930
P36	11.623532	8.356903	3.401764
P37	1.500628	11.664473	5.518221
P38	3.823702	10.001182	3.385781
P39	0.000911	10.008524	5.516404
P40	2.327304	11.662133	3.382270
P41	6.129459	11.662229	5.544904
P42	8.474277	10.000533	3.430065
P43	4.629029	10.004595	5.532307
P44	6.977411	11.662317	3.417541
P45	10.799589	11.665295	5.549775
P46	13.126989	10.018828	3.381940
P47	9.311590	9.997077	5.560356
P48	11.615323	11.665106	3.405781
Au1	7.559843	5.052359	7.116054
O1	8.904969	4.353639	9.763296
N1	8.281915	5.176609	9.142904
O2	8.482420	6.526268	9.651311
H1	7.885777	7.059819	9.081773

FigureS4d

P1	1.989830	2.207990	5.493684
P2	4.319545	0.548207	3.378913
P3	0.498907	0.546137	5.505665
P4	2.809189	2.197910	3.358265
P5	6.617287	2.204236	5.550044
P6	8.968454	0.550207	3.433171
P7	5.121595	0.543502	5.523365
P8	7.479030	2.216030	3.430796
P9	11.315914	2.201757	5.538499
P10	13.612521	0.548435	3.377444
P11	9.812924	0.548409	5.558843
P12	12.108762	2.204443	3.398525
P13	1.985016	5.528707	5.502427
P14	4.300717	3.864779	3.367174
P15	0.493609	3.865481	5.517733
P16	2.801588	5.527554	3.367353
P17	6.511388	5.524646	5.423963
P18	8.985942	3.865545	3.482799
P19	5.074178	3.826852	5.518264

P20	7.504996	5.528468	3.371462
P21	11.306011	5.529474	5.592872
P22	13.586989	3.878494	3.397561
P23	9.806946	3.861462	5.618875
P24	12.081073	5.530095	3.438952
P25	1.989587	8.849351	5.493974
P26	4.301068	7.189702	3.367464
P27	0.493445	7.191955	5.515067
P28	2.811719	8.857475	3.360067
P29	6.619173	8.851869	5.547817
P30	8.984179	7.191554	3.484098
P31	5.079503	7.225835	5.518063
P32	7.479300	8.841492	3.429059
P33	11.323440	8.858198	5.528635
P34	13.590446	7.178640	3.393887
P35	9.811397	7.201669	5.617830
P36	12.115543	8.855716	3.389627
P37	1.996771	12.168961	5.501568
P38	4.320299	10.507985	3.379932
P39	0.499521	10.511826	5.504175
P40	2.824511	12.167956	3.369526
P41	6.622070	12.168314	5.542472
P42	8.968407	10.507511	3.431112
P43	5.122452	10.512215	5.523484
P44	7.473716	12.168719	3.420230
P45	11.307798	12.169416	5.532092
P46	13.617075	10.513924	3.374471
P47	9.816947	10.507448	5.555031
P48	12.116261	12.169446	3.389906
Au1	7.807396	5.537275	7.278346
O1	8.710326	5.464244	9.082080
H1	8.835578	6.389365	9.365358
N1	6.400392	5.434253	10.706793
O2	5.599987	4.630005	10.269336
O3	6.809183	5.647899	11.829417

FigureS4e

P1	1.800085	2.283475	5.450984
P2	4.136154	0.625960	3.335128
P3	0.307446	0.622644	5.441093
P4	2.633348	2.282691	3.320198
P5	6.431644	2.282731	5.513593
P6	8.778898	0.639497	3.371456
P7	4.937924	0.618293	5.478517
P8	7.279530	2.299241	3.387365
P9	11.177131	2.247876	5.514557
P10	13.438913	0.615687	3.307517

P11	9.622636	0.634173	5.500385
P12	11.932596	2.279003	3.363790
P13	1.795491	5.608251	5.453628
P14	4.128849	3.953070	3.339573
P15	0.305706	3.944921	5.453234
P16	2.632339	5.617434	3.326220
P17	6.346797	5.583924	5.477715
P18	8.771389	3.960926	3.370158
P19	4.877797	3.908560	5.495957
P20	7.276943	5.596685	3.351539
P21	11.162020	5.629288	5.487669
P22	13.426291	3.940094	3.324064
P23	9.684254	3.964543	5.500863
P24	11.926293	5.601312	3.334276
P25	1.797571	8.929609	5.451463
P26	4.140026	7.264533	3.383411
P27	0.302474	7.269838	5.454287
P28	2.639576	8.930213	3.324988
P29	6.451206	8.925864	5.508851
P30	8.771948	7.253131	3.398955
P31	4.895790	7.307071	5.542029
P32	7.275647	8.914469	3.376500
P33	11.124101	8.939681	5.474253
P34	13.420250	7.267716	3.323144
P35	9.622368	7.265318	5.531479
P36	11.923042	8.927618	3.332365
P37	1.803501	12.244502	5.457004
P38	4.144240	10.583094	3.345267
P39	0.304268	10.588438	5.451581
P40	2.643054	12.240673	3.328600
P41	6.442958	12.247685	5.497497
P42	8.771476	10.582036	3.365605
P43	4.951570	10.583865	5.489537
P44	7.284217	12.252413	3.370748
P45	11.113186	12.250393	5.472919
P46	13.424327	10.587873	3.319424
P47	9.619440	10.589121	5.490472
P48	11.927009	12.247590	3.329495
Au1	8.028920	4.937219	7.032682
O1	8.235320	4.608800	9.405253
H1	8.925889	5.218084	9.758725
N1	7.120220	4.627565	10.599474
O2	6.107489	4.071741	10.271965
O3	7.440249	5.298104	11.558459

FigureS4f

P1	1.723479	2.316610	5.433290
P2	4.058772	0.664009	3.323602

P3	0.229904	0.655664	5.410974
P4	2.555934	2.318716	3.304212
P5	6.356033	2.347719	5.515567
P6	8.693267	0.677051	3.347407
P7	4.858774	0.659110	5.465303
P8	7.197370	2.339232	3.376516
P9	11.128165	2.265844	5.464922
P10	13.373126	0.637790	3.273271
P11	9.548148	0.667047	5.468555
P12	11.878559	2.309737	3.312670
P13	1.719602	5.645410	5.435176
P14	4.043565	3.991688	3.305296
P15	0.230753	3.979036	5.424021
P16	2.545925	5.654827	3.306894
P17	6.285019	5.630686	5.483909
P18	8.694812	3.989673	3.269285
P19	4.802114	3.968552	5.458727
P20	7.196280	5.623732	3.335629
P21	11.112060	5.675061	5.450136
P22	13.373263	3.974645	3.289056
P23	9.680646	3.972920	5.384261
P24	11.872991	5.636101	3.300315
P25	1.717840	8.970373	5.423720
P26	4.040691	7.315435	3.351608
P27	0.226763	7.308249	5.419746
P28	2.539451	8.984169	3.292062
P29	6.353005	8.953046	5.489632
P30	8.686914	7.285260	3.348880
P31	4.787779	7.349641	5.503535
P32	7.188062	8.946026	3.358293
P33	11.035345	8.957243	5.440343
P34	13.362123	7.310467	3.284650
P35	9.542112	7.290735	5.472005
P36	11.847824	8.956772	3.299569
P37	1.722970	12.277639	5.435973
P38	4.059438	10.623990	3.323233
P39	0.218803	10.624905	5.433534
P40	2.561634	12.280005	3.307834
P41	6.363381	12.288506	5.479086
P42	8.681260	10.614254	3.342604
P43	4.870377	10.624843	5.467515
P44	7.198840	12.289965	3.352278
P45	11.035427	12.279040	5.444828
P46	13.340815	10.620235	3.300961
P47	9.539950	10.617565	5.461236
P48	11.846736	12.281369	3.301340
Au1	7.900716	4.484029	6.897194
O1	8.411342	4.227088	9.665073

H1	9.114490	4.785231	10.069126
N1	7.297957	4.390427	10.617324
O2	6.290427	3.833359	10.259063
O3	7.554205	5.062068	11.600012

FigureS4g

P1	1.921024	1.651986	5.564419
P2	2.740864	1.640985	3.426869
P3	6.577035	1.652042	5.568118
P4	7.398235	1.656992	3.429089
P5	11.221155	1.651088	5.584535
P6	12.052390	1.651847	3.448883
P7	1.917732	4.972244	5.575555
P8	4.229704	3.308347	3.419840
P9	0.425054	3.311115	5.574097
P10	2.746571	4.981154	3.440344
P11	6.494685	4.953616	5.465058
P12	8.900291	3.315609	3.424237
P13	5.037465	3.277998	5.561191
P14	7.422557	4.978548	3.364369
P15	11.237680	4.980135	5.582211
P16	13.543304	3.313507	3.441561
P17	9.723134	3.331493	5.565246
P18	12.046817	4.974626	3.443963
P19	1.923236	8.291028	5.578036
P20	4.255111	6.626253	3.467743
P21	0.425313	6.634481	5.576890
P22	2.765602	8.288412	3.446174
P23	6.594649	8.298822	5.594142
P24	8.908217	6.639635	3.454317
P25	5.063497	6.658204	5.632862
P26	7.397728	8.288347	3.449228
P27	11.234208	8.303529	5.564355
P28	13.548753	6.632702	3.439290
P29	9.735255	6.640695	5.593919
P30	12.060562	8.298869	3.429811
P31	1.928156	11.611404	5.576763
P32	4.260919	9.947929	3.454792
P33	0.430656	9.952835	5.573208
P34	2.760410	11.604124	3.443809
P35	6.580138	11.616157	5.577493
P36	8.892485	9.952293	3.440136
P37	5.087512	9.952174	5.592122
P38	7.397786	11.613774	3.438968
P39	11.219120	11.618888	5.568309
P40	13.558341	9.958419	3.434742
P41	9.729881	9.954338	5.572791
P42	12.056503	11.614014	3.434174

P43	4.248434	13.269992	3.436037
P44	0.429848	13.269584	5.570978
P45	8.895002	13.275588	3.432237
P46	5.079900	13.271145	5.572090
P47	13.552791	13.272354	3.436437
P48	9.719180	13.275792	5.568091
Au1	7.971476	4.664093	7.332915
C1	9.041918	4.344422	9.109106
H1	8.520823	3.542989	9.650764
H2	10.076404	4.047796	8.896475
H3	9.026981	5.271204	9.696472
N1	6.530484	6.244613	8.572855
O1	6.105795	5.885704	9.606569

FigureS4h

P1	1.929394	1.986830	5.550820
P2	2.752259	1.976334	3.414025
P3	6.586268	1.981399	5.563638
P4	7.410870	1.989061	3.425848
P5	11.264147	1.973816	5.541947
P6	12.079275	1.979671	3.404401
P7	1.929037	5.306734	5.559438
P8	4.241172	3.643892	3.412759
P9	0.434116	3.646401	5.556466
P10	2.751739	5.311659	3.423771
P11	6.481720	5.293886	5.442749
P12	8.917623	3.642545	3.422623
P13	5.041755	3.604631	5.558607
P14	7.439367	5.306682	3.361365
P15	11.265877	5.305525	5.526269
P16	13.567466	3.646746	3.406261
P17	9.766521	3.645625	5.553666
P18	12.066148	5.307116	3.386775
P19	1.933082	8.626884	5.557229
P20	4.253518	6.966761	3.439851
P21	0.436004	6.969264	5.558440
P22	2.764981	8.633576	3.423306
P23	6.593327	8.625418	5.586303
P24	8.917205	6.972523	3.443296
P25	5.051556	6.998730	5.595469
P26	7.409881	8.623414	3.446124
P27	11.257320	8.632788	5.545712
P28	13.563911	6.971022	3.409526
P29	9.763075	6.963499	5.572416
P30	12.072667	8.635717	3.408424
P31	1.937962	11.946589	5.562851
P32	4.269405	10.285652	3.441004
P33	0.440403	10.288652	5.556358

P34	2.772998	11.944671	3.430198
P35	6.583789	11.945750	5.581080
P36	8.904096	10.287288	3.440867
P37	5.089643	10.283764	5.581714
P38	7.409967	11.949611	3.445280
P39	11.241042	11.948903	5.558043
P40	13.569406	10.297335	3.414880
P41	9.752235	10.283665	5.568493
P42	12.065989	11.951326	3.418829
P43	4.265293	13.607177	3.434794
P44	0.439176	13.604029	5.554961
P45	8.906053	13.611377	3.442626
P46	5.084682	13.603344	5.575943
P47	13.568945	13.604706	3.416496
P48	9.747807	13.611112	5.572707
Au1	7.950645	5.113776	7.224685
C1	9.762796	5.048036	9.344393
H1	9.672507	4.010220	9.686636
H2	10.584524	5.134759	8.622958
H3	9.953055	5.703973	10.204305
N1	7.870180	5.950264	9.221389
O1	7.250116	5.537247	10.146027

FigureS4i

P1	1.906034	1.644817	5.546556
P2	2.735889	1.635798	3.410588
P3	6.557856	1.644942	5.589473
P4	7.388890	1.651520	3.452713
P5	11.243973	1.636379	5.556882
P6	12.046380	1.641019	3.413902
P7	1.902604	4.966647	5.552823
P8	4.223594	3.303972	3.421368
P9	0.411066	3.303753	5.548946
P10	2.727962	4.966588	3.418070
P11	6.439193	4.969162	5.471222
P12	8.895382	3.302324	3.470938
P13	5.009697	3.264839	5.578176
P14	7.417185	4.965278	3.399711
P15	11.247505	4.968298	5.563137
P16	13.537548	3.305013	3.412111
P17	9.746758	3.306828	5.599821
P18	12.038985	4.965093	3.416762
P19	1.905820	8.287544	5.546182
P20	4.224071	6.628968	3.424212
P21	0.410249	6.628942	5.549483
P22	2.736143	8.297873	3.410527
P23	6.555423	8.288275	5.592039
P24	8.894699	6.630234	3.474914

P25	5.004890	6.670650	5.580775
P26	7.387710	8.280890	3.456421
P27	11.241193	8.294211	5.559664
P28	13.535542	6.627158	3.412930
P29	9.742967	6.624162	5.603294
P30	12.044451	8.291150	3.416099
P31	1.912890	11.606407	5.555042
P32	4.245360	9.947107	3.432414
P33	0.414617	9.949510	5.547300
P34	2.749855	11.606776	3.421323
P35	6.556419	11.607162	5.584470
P36	8.882289	9.943626	3.449018
P37	5.056646	9.950282	5.578449
P38	7.387598	11.606387	3.449084
P39	11.224085	11.605845	5.557685
P40	13.543165	9.951706	3.408379
P41	9.732122	9.943189	5.577647
P42	12.040397	11.605826	3.413645
P43	4.245154	13.266957	3.432240
P44	0.414567	13.263026	5.546566
P45	8.882952	13.268419	3.446967
P46	5.057287	13.264624	5.577962
P47	13.543691	13.259507	3.407250
P48	9.733300	13.269367	5.575712
Au1	7.844900	4.962419	7.295485
C1	10.119786	4.518968	9.418913
H1	10.137071	3.419283	9.501229
H2	10.747456	4.819641	8.568065
H3	10.477077	4.963373	10.357872
N1	8.737951	4.959137	9.186154
O1	8.065730	5.247388	10.204090

FigureS4j

P1	1.928005	1.641285	5.597525
P2	2.755457	1.633463	3.466250
P3	6.576435	1.640698	5.632637
P4	7.409574	1.650576	3.504410
P5	11.254326	1.637832	5.609917
P6	12.065816	1.634086	3.477764
P7	1.924726	4.962177	5.606369
P8	4.244720	3.300491	3.475760
P9	0.431726	3.299607	5.610187
P10	2.749992	4.964423	3.476017
P11	6.473737	4.960400	5.519293
P12	8.910044	3.307254	3.539572
P13	5.033153	3.266735	5.621803
P14	7.429414	4.965646	3.443777
P15	11.239552	4.964168	5.657946

P16	13.540859	3.309367	3.485027
P17	9.726242	3.308916	5.679976
P18	12.032301	4.960478	3.515749
P19	1.928322	8.282970	5.599605
P20	4.250738	6.623712	3.483448
P21	0.431685	6.624708	5.611767
P22	2.760191	8.289684	3.469676
P23	6.581211	8.287000	5.636650
P24	8.913061	6.623391	3.539178
P25	5.038511	6.659840	5.630596
P26	7.410326	8.278042	3.506376
P27	11.253611	8.292998	5.610851
P28	13.538456	6.614143	3.487228
P29	9.733852	6.625525	5.678336
P30	12.062974	8.289680	3.478301
P31	1.937957	11.601950	5.606591
P32	4.267420	9.941234	3.486622
P33	0.437896	9.945814	5.598864
P34	2.771151	11.601019	3.477402
P35	6.579345	11.604332	5.628101
P36	8.904340	9.941512	3.500097
P37	5.080909	9.945752	5.626346
P38	7.409441	11.604549	3.497573
P39	11.243994	11.606120	5.603820
P40	13.569982	9.946271	3.464394
P41	9.752669	9.944956	5.623638
P42	12.070446	11.603178	3.467142
P43	4.264624	13.263239	3.483186
P44	0.437877	13.258200	5.597594
P45	8.904376	13.267878	3.499399
P46	5.078198	13.260401	5.622758
P47	13.573492	13.256874	3.462251
P48	9.752735	13.267174	5.622704
Au1	8.003526	4.894034	7.347876
C1	9.311850	4.819612	8.997714
H1	9.062372	3.914833	9.567330
H2	10.357481	4.787438	8.667353
H3	9.134503	5.715729	9.606607
N1	5.837626	6.344316	9.346975
O1	6.155968	5.191357	9.084195
O2	5.718966	4.156046	9.568755

FigureS4k

P1	1.691907	1.993296	5.543745
P2	2.507064	1.976490	3.409664
P3	6.338327	1.996876	5.571285
P4	7.177072	1.998719	3.448215
P5	11.035428	1.977091	5.569119

P6	11.825141	1.989291	3.429118
P7	1.690568	5.313517	5.548832
P8	4.000293	3.648374	3.415221
P9	0.195192	3.652919	5.554283
P10	2.507036	5.316483	3.414376
P11	6.208898	5.308179	5.465108
P12	8.677317	3.661810	3.495132
P13	4.775104	3.613815	5.555486
P14	7.191002	5.311694	3.371339
P15	11.026453	5.317116	5.579429
P16	13.315590	3.655202	3.428758
P17	9.500457	3.688170	5.650332
P18	11.814148	5.312708	3.428181
P19	1.694164	8.633629	5.545252
P20	4.005199	6.977746	3.425144
P21	0.195675	6.975247	5.553566
P22	2.510701	8.649234	3.410463
P23	6.344795	8.632770	5.574241
P24	8.678298	6.969373	3.490846
P25	4.780235	7.010052	5.565050
P26	7.177845	8.630895	3.450057
P27	11.026420	8.649702	5.563431
P28	13.312962	6.973732	3.430191
P29	9.501523	6.950673	5.640090
P30	11.825977	8.642475	3.426968
P31	1.699692	11.952309	5.559891
P32	4.024550	10.293577	3.433219
P33	0.199892	10.294400	5.553190
P34	2.528170	11.951652	3.429875
P35	6.336460	11.953732	5.580069
P36	8.669870	10.296073	3.452477
P37	4.841986	10.291413	5.570847
P38	7.173712	11.957184	3.452550
P39	11.001502	11.955527	5.564775
P40	13.334339	10.304060	3.420551
P41	9.512391	10.290001	5.576210
P42	11.829379	11.955132	3.428328
P43	4.021826	13.611057	3.435297
P44	0.201865	13.613183	5.555868
P45	8.675995	13.612136	3.456801
P46	4.841744	13.617574	5.570807
P47	13.340831	13.599338	3.420739
P48	9.514164	13.622857	5.583136
Au1	7.990106	5.223850	7.090351
C1	9.470505	5.515071	9.963064
H1	10.286332	5.488440	9.205200
H2	9.331672	6.462300	10.511657
H3	9.190876	4.557970	10.447010

N1	7.127300	5.785314	9.602609
O1	6.492178	4.746369	9.632906
O2	6.689870	6.938161	9.604135

FigureS4l

P1	1.671952	1.521239	5.423517
P2	2.494650	1.508629	3.283220
P3	6.311485	1.520860	5.475457
P4	7.157951	1.529548	3.343699
P5	11.012686	1.510063	5.449914
P6	11.808827	1.516522	3.301057
P7	1.665844	4.842822	5.431619
P8	3.980997	3.179813	3.297926
P9	0.175255	3.179238	5.432751
P10	2.485666	4.843854	3.292314
P11	6.169812	4.843411	5.333445
P12	8.663288	3.181784	3.369794
P13	4.751511	3.134001	5.460390
P14	7.179818	4.843050	3.271770
P15	11.030405	4.843063	5.452860
P16	13.300489	3.180785	3.294335
P17	9.523802	3.192016	5.496648
P18	11.806012	4.844439	3.298114
P19	1.673368	8.164667	5.425538
P20	3.984990	6.504434	3.299306
P21	0.176458	6.506970	5.433129
P22	2.499855	8.175888	3.286184
P23	6.317469	8.168872	5.473806
P24	8.661677	6.504285	3.365773
P25	4.758522	6.554172	5.462457
P26	7.157428	8.157620	3.340198
P27	11.007711	8.175273	5.455674
P28	13.300760	6.507556	3.294873
P29	9.518504	6.489624	5.495575
P30	11.806455	8.168864	3.307486
P31	1.680660	11.483035	5.435990
P32	4.012224	9.823205	3.307501
P33	0.182510	9.826132	5.431767
P34	2.515492	11.482215	3.298763
P35	6.317152	11.484608	5.469724
P36	8.652980	9.820672	3.337619
P37	4.817545	9.827166	5.457735
P38	7.157342	11.483709	3.335224
P39	10.987604	11.481609	5.452499
P40	13.305944	9.829662	3.292526
P41	9.493417	9.819419	5.471465
P42	11.802427	11.482350	3.305058
P43	4.009553	13.143595	3.306138
P44	0.181645	13.139341	5.431651

P45	8.652376	13.146426	3.339602
P46	4.814881	13.140079	5.456908
P47	13.306791	13.134642	3.291728
P48	9.497469	13.147312	5.471716
Au1	7.676353	4.842065	7.030687
C1	10.060511	5.729529	10.527247
H1	11.025723	5.568867	10.028322
H2	10.034436	6.695758	11.037552
H3	9.867901	4.899106	11.217925
N1	9.007141	5.740517	9.499013
O1	8.875032	4.617101	8.846193
O2	8.353660	6.763296	9.238386
